# Mutation in *OsFWL7* Affects Cadmium and Micronutrient Metal Accumulation in Rice

**DOI:** 10.3390/ijms222212583

**Published:** 2021-11-22

**Authors:** Qingsong Gao, Lei Liu, Haiying Zhou, Xi Liu, Wei Li, Yu Min, Yurong Yan, Jianhui Ji, Hao Zhang, Xiangxiang Zhao

**Affiliations:** 1 Jiangsu Collaborative Innovation Center of Regional Modern Agriculture & Environmental Protection/Jiangsu Key Laboratory for Eco-Agricultural Biotechnology around Hongze Lake, Huaiyin Normal University, Huai’an 223300, China; qsgao@hytc.edu.cn (Q.G.); hyzhou2018@163.com (H.Z.); 8201811078@hytc.edu.cn (X.L.); 18344870838@139.com (W.L.); m2567468323@163.com (Y.M.); y19852599546@163.com (Y.Y.); jijianhui@hytc.edu.cn (J.J.); 2 Jiangsu Key Laboratory of Crop Genetics and Physiology/Jiangsu Key Laboratory of Crop Cultivation and Physiology, Jiangsu Co-Innovation Center for Modern Production Technology of Grain Crops, Yangzhou University, Yangzhou 225009, China; liulei128478@163.com

**Keywords:** rice, *FW2.2*-like gene, cadmium, micronutrient metal, protein interaction, membrane microdomain

## Abstract

Micronutrient metals, such as Mn, Cu, Fe, and Zn, are essential heavy metals for plant growth and development, while Cd is a nonessential heavy metal that is highly toxic to both plants and humans. Our understanding of the molecular mechanisms underlying Cd and micronutrient metal accumulation in plants remains incomplete. Here, we show that *OsFWL7*, an *FW2.2*-like (*FWL*) family gene in *Oryza sativa*, is preferentially expressed in the root and encodes a protein localized to the cell membrane. The *osfwl7* mutation reduces both the uptake and the root-to-shoot translocation of Cd in rice plants. Additionally, the accumulation of micronutrient metals, including Mn, Cu, and Fe, was lower in *osfwl7* mutants than in the wildtype plants under normal growth conditions. Moreover, the *osfwl7* mutation affects the expression of several heavy metal transporter genes. Protein interaction analyses reveal that rice FWL proteins interact with themselves and one another, and with several membrane microdomain marker proteins. Our results suggest that *OsFWL7* is involved in Cd and micronutrient metal accumulation in rice. Additionally, rice FWL proteins may form oligomers and some of them may be located in membrane microdomains.

## 1. Introduction

Micronutrient metals, such as Mn, Cu, Fe, and Zn, are essential heavy metals for plant growth and development [1]. In contrast, Cd is a nonessential heavy metal that is highly toxic. It disrupts plant growth and development, thereby substantially reducing crop yield. Moreover, upon entering the food chain, it threatens public health [2,3]. Therefore, elucidating the molecular mechanisms underlying plant Cd and micronutrient metal accumulation will aid the development of crop varieties with enhanced nutrient uptake and reduced Cd accumulation.

Rice (*Oryza sativa* L.) is one of the most important cereal crops worldwide. Since Cd is nonessential for growth, plants may not possess a specific transporter for this metal, and Cd likely enters rice cells via transporters for essential metals. *OsNramp5* is mainly expressed in rice roots and encodes a plasma membrane-localized transporter [4,5]. The loss-of-function mutation of this gene results in an extremely low Cd accumulation in roots, shoots, and grains, suggesting that OsNramp5 is a major transporter for Cd uptake in rice [4,6]. However, OsNramp5 also acts as a Mn transporter, and the knockout of this gene significantly reduces plant growth [4]. Cd taken up from soil is sequestered into the vacuoles of root cells, and OsHMA3—a P_1B_-type ATPase—plays an important role in this process [7,8]. This transporter is localized to the tonoplast of all root cells, and the expression of gene encoding OsHMA3 is unaffected by Cd treatment. The loss of protein function decreases the vacuolar sequestration of Cd in roots, resulting in high root-to-shoot translocation [7,8,9,10]. However, the overexpression of this gene also increases the Zn content of roots, suggesting that OsHMA3 may originally be a Zn transporter [11]. The root-to-shoot transportation of Cd is mediated by OsHMA2, a homolog of OsHMA3 [12,13,14]. OsHMA2 is also a Zn transporter [12]. Additionally, the plasma membrane-localized transporters OsIRT1 and OsIRT2 are involved in Fe uptake [15]. Cu is mainly taken up by COPT transporters, such as COPT1 and COPT5, and loaded into the xylem for transportation by OsHMA5 [16,17,18]. Unfortunately, despite great research progress, our understanding of the molecular mechanisms underlying Cd and micronutrient metal accumulation in rice remains incomplete.

FW2.2 is the key regulator of tomato fruit size and weight, and a negative regulator of cell proliferation during fruit development [19,20]. The FW2.2 protein harbors an uncharacterized placenta-specific 8 domain, and is localized to the plasma membrane [21,22]. *FW2.2*-like (*FWL*) genes have been characterized in various plant species and reported to perform diverse functions [23,24,25]. *Cell Number Regulator1* (*CNR1*), an *FWL* gene in maize, negatively regulates plant and organ size by controlling cell division [22]. Similarly, *Physalis floridana CNR1* negatively affects the size of multiple organs by altering the cell number [26]. Additionally, the soybean *FWL* gene *GmFWL1* plays a pivotal role in nodulation [27]. RNA interference-mediated knockdown of *GmFWL1* significantly reduced the nodule number. Interestingly, GmFWL1 is a plasma membrane microdomain-associated protein [28]. In addition, FWL proteins play important roles in heavy metal homeostasis. *FWL* homologs in Arabidopsis have been named *Plant Cadmium Resistance* genes (*PCR*s). In particular, *AtPCR1* overexpression enhances Cd tolerance in both yeast and Arabidopsis by reducing its accumulation [29]. AtPCR2 forms homo-oligomers in the plasma membrane and mediates Zn transport in Arabidopsis [30]. Additionally, overexpression of the common wheat *FWL* gene *TaCNR2* and the diploid wheat *FWL* gene *TuCNR10* in Arabidopsis and rice improved the tolerance and translocation of Cd, Zn, and Mn [31,32].

Previously, eight *FWL* genes (*OsFWL1*–*OsFWL8*) were identified in the rice genome [33]. Among these, *OsCNR1*/*FWL1* determines rice grain width and weight by influencing cell division and expansion in the glumes [34]. Changes in *OsFWL1* and *OsFWL2* expression affect Cd tolerance and accumulation [35]. OsFWL4 can form homo-oligomers in the plasma membrane and is involved in the root-to-shoot transportation of Cd [36]. Additionally, this protein acts as a negative regulator of rice tiller number [37]. OsPCR1/FWL5 is localized to the plasma membrane as an oligomer and affects the grain weight and Zn content [38].

In this study, we aimed to characterize the role of *OsFWL7* (*LOC_Os03g61500*) in heavy metal accumulation. Cd treatment induced significant *OsFWL7* expression in roots. Moreover, the *osfwl7* mutation reduced the accumulation of Cd and micronutrient metals, including Mn, Cu, and Fe, in rice. We also found that the rice FWL proteins self-interact and interact with one another, and that some of them may be located in membrane microdomains.

## 2. Results

### 2.1. Characterization of OsFWL7

**Figure 1:**
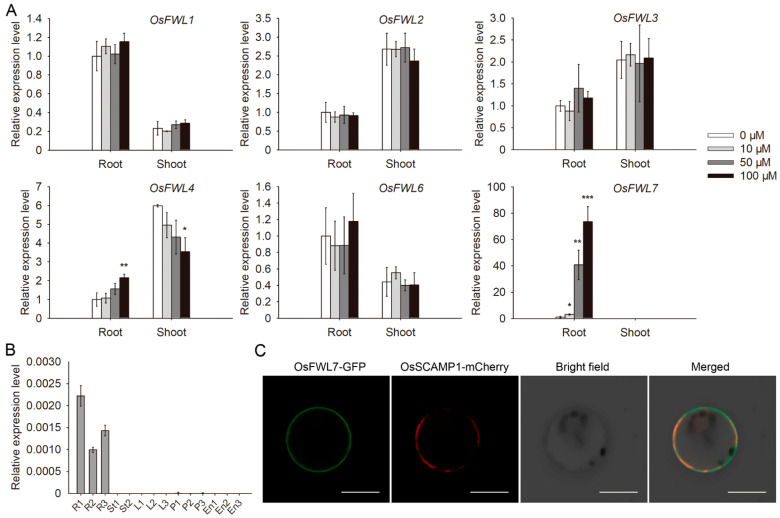
Gene expression profiles and subcellular localization of OsFWL7. (**A**) Expression patterns of six rice *FWL* genes under treatment with Cd of different concentrations, as determined using RT-qPCR. The rice *Actin1* gene was used for normalization of gene expression. Error bars indicate the standard deviation of three biological replicates. * *p* < 0.05, ** *p* < 0.01, *** *p* < 0.001. (**B**) *OsFWL7* expression patterns in 14 tissue samples of *Oryza sativa* L. ssp. *japonica* variety Zhonghua 11, as determined using RT-qPCR. The tissues used were as follows: seedling, tillering, and heading stage roots (R1–R3); jointing and heading stage stems (St1 and St2); seedling, tillering, and heading stage leaves (L1–L3); 5-, 15-, and 20-cm panicles (P1–P3); and endosperms 5, 14, and 21 days after pollination (En1–En3). Error bars indicate the standard deviation of three technical replicates. (**C**) Subcellular localization of OsFWL7. The *OsSCAMP1*-mCherry construct was used as the plasma membrane marker. The GFP fluorescence, mCherry fluorescence, bright field, and merged images are shown. Bar = 10 μm.

Previous studies have reported that the expression of several rice *FWL* genes may enhance the Cd tolerance of sensitive yeast cells [29,36]. To test whether the *FWL* genes are involved in Cd response in rice, the expression patterns of six genes (*OsFWL1–OsFWL4*, *OsFWL6*, and *OsFWL7*) under Cd exposure at different concentrations were examined using reverse-transcription quantitative PCR (RT-qPCR). *Actin1*, a commonly used reference gene in rice [39,40], was used for data normalization. The expression of only two genes, namely *OsFWL4* and *OsFWL7*, was found to be significantly altered (Figure 1A). The *OsFWL4* transcript level increased in roots but decreased in shoots following exposure to 100 μM Cd, which is consistent with previous reports [36]. Cd exposure markedly induced *OsFWL7* expression in the roots (Figure 1A). However, the *OsFWL7* transcript level was below the limit of detection in shoots both under normal conditions and all Cd treatments. Next, we examined the expression profile of this gene in different tissues and observed that *OsFWL7* was predominantly expressed in the roots (Figure 1B). The expression patterns of *OsFWL7* in different tissues and under Cd treatments were also analyzed using rice ubiquitin gene (*LOC_Os03g13170*) as a reference [41], and similar results were obtained (Figure S1).

Protein sequence analysis using TMHMM Server v. 2.0 (https://services.healthtech.dtu.dk/service.php?TMHMM-2.0) predicted a transmembrane helix in the 50–72 region of OsFWL7 (Figure S2). The protein was fused with GFP to determine its subcellular localization. A known plasma membrane protein, OsSCAMP1 [42], was fused with mCherry and used as the marker. GFP fluorescence was detected only in the plasma membrane of rice protoplasts and co-localized with mCherry fluorescence (Figure 1C), suggesting that OsFWL7 is localized to the cell membrane.

### 2.2. osfwl7 Mutants Are Less Sensitive to Cd

We have previously designed two target sites (Osfwl7a and Osfwl7b) in the *OsFWL7* gene for clustered regularly interspaced short palindromic repeats (CRISPR)/CRISPR-associated protein 9 (Cas9)-mediated genome editing [37]. Both the target sites were located in the second exon of the gene (Figure S3A). For Osfwl7a target, 13 T0 mutants were obtained, among which two were homozygotes [37]. For Osfwl7b target, 12 T0 mutants were obtained and five of them were homozygotes. We selected one homozygous mutant of each target for further analysis, and designated them *osfwl7a* and *osfwl7b*, respectively. Both mutant lines harbored a 1 bp insertion in the coding region (Figure S3B,C), which caused frame-shift mutations in the gene. The grain yield per plant and plant height of *osfwl7* mutants grown under normal field conditions were not significantly different from those of the wildtype (WT) (Figure S4).

**Figure 2:**
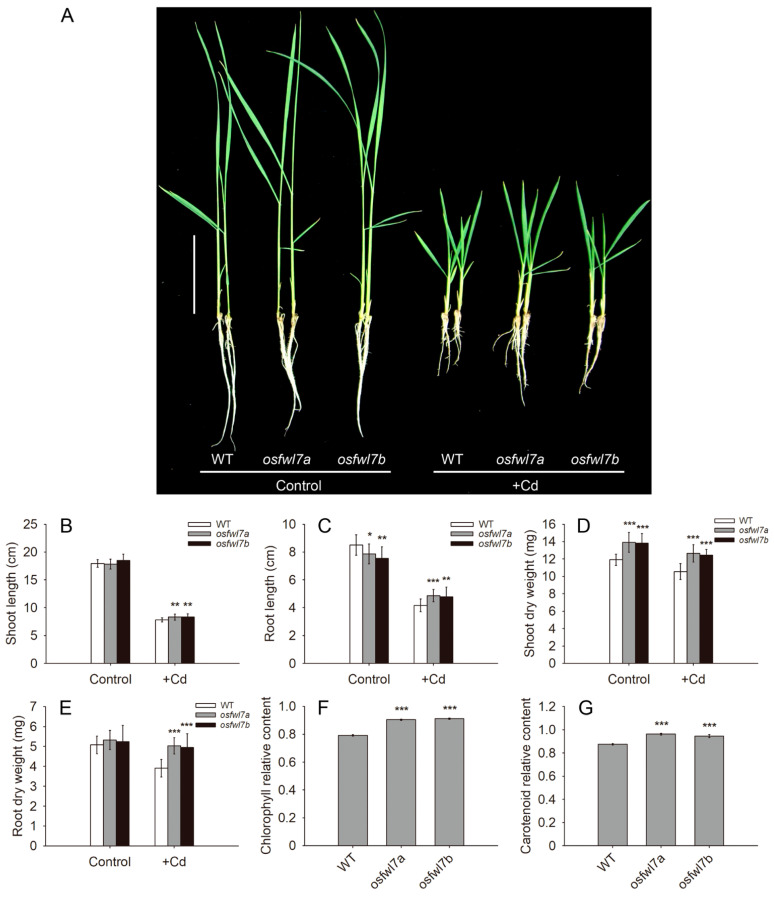
Performance of the wildtype (WT) and *osfwl7* mutants under Cd stress. (**A**) WT and mutant plants grown under normal conditions and in the presence of 50 μM Cd for 10 days, bar = 5 cm. (**B**–**E**) Shoot length (**B**), root length (**C**), shoot dry weight (**D**), and root dry weight (**E**) of the WT and mutants, *n* = 15. Error bars indicate standard deviation. (**F**) Relative chlorophyll content of the WT and mutants (chlorophyll content under Cd stress/chlorophyll content under normal condition). (**G**) Relative carotenoid content of the WT and mutants (carotenoid content under Cd stress/carotenoid content under normal condition). Error bars in (**F**,**G**) indicate the standard deviation of three biological replicates. * *p* < 0.05, ** *p* < 0.01, *** *p* < 0.001.

To investigate the biological function of *OsFWL7* in Cd response, the *osfwl7a* and *osfwl7b* mutants were treated with 50 μM Cd. Under normal growth conditions, the shoot length of *osfwl7* mutants was similar to that of the WT, but the root length was slightly lower (Figure 2A–C). Cd markedly inhibited shoot and root elongation in both WT and mutant plants (Figure 2A–C). However, the shoots and roots of *osfwl7* mutants were significantly longer than those of the WT under Cd stress (Figure 2A–C). The shoot dry weight of *osfwl7* mutants was greater than that of the WT, both under normal conditions and Cd stress (Figure 2D). The root dry weight of *osfwl7* mutants did not evidently differ from that of the WT under normal conditions, but was markedly greater under Cd stress (Figure 2E). Among leaf pigments, the chlorophyll and carotenoid contents of both WT and mutant plants decreased following Cd treatment. However, this decrease was significantly smaller in *osfwl7* mutants (Figure 2F,G). Taken together, these results suggest that *osfwl7* mutants are less sensitive to Cd stress.

### 2.3. OsFWL7 Is Involved in Cd Uptake and Translocation

**Figure 3:**
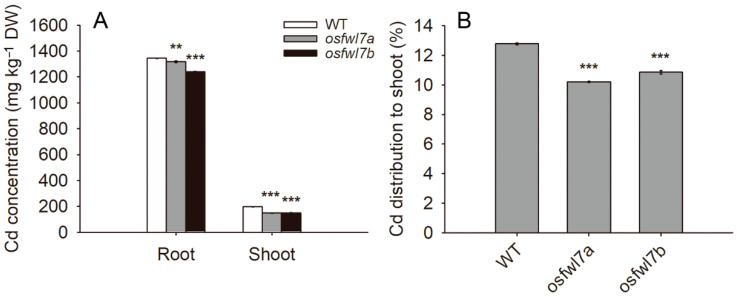
Effect of *osfwl7* mutation on Cd accumulation and translocation. (**A**) Measurement of Cd levels in the wildtype (WT) and *osfwl7* mutants grown in the presence of 50 μM Cd for 10 days. (**B**) The percentage of Cd distributed to the shoot. Error bars indicate the standard deviation of three biological replicates. ** *p* < 0.01, *** *p* < 0.001.

To test whether the *osfwl7* mutation affects Cd accumulation, Cd concentrations in the roots and shoots of WT and mutant plants under Cd treatment were determined. The Cd concentrations in both roots and shoots were significantly lower in the *osfwl7* mutants than in the WT (Figure 3A). Additionally, the proportion of Cd distributed to the shoots was lower in *osfwl7* mutants (Figure 3B). These results suggest that *OsFWL7* is involved in both Cd uptake and translocation. 

### 2.4. osfwl7 Mutation Affects Micronutrient Metal Accumulation in Rice

**Figure 4:**
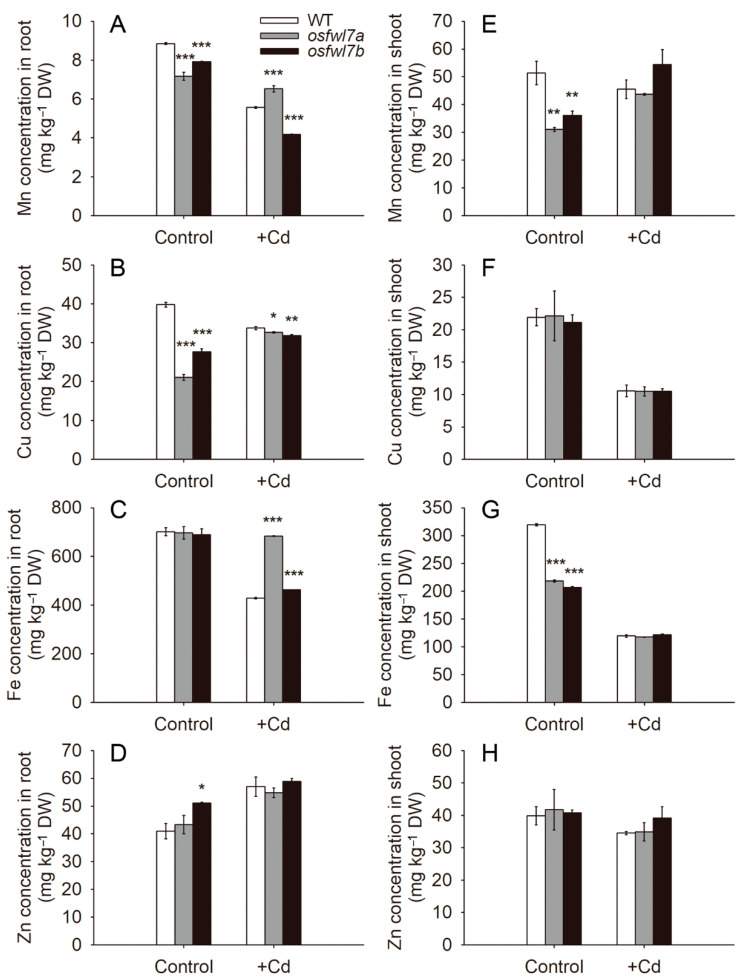
Effect of the *osfwl7* mutation on micronutrient metal accumulation. (**A**–**D**) Measurement of Mn (**A**), Cu (**B**), Fe (**C**), and Zn (**D**) levels in roots of the wildtype (WT) and mutants grown under normal conditions and in the presence of 50 μM Cd for 10 days. (**E**–**H**) Measurement of Mn (**E**), Cu (**F**), Fe (**G**), and Zn (**H**) levels in the shoots of WT and mutants. Error bars indicate the standard deviation of three biological replicates. * *p* < 0.05, ** *p* < 0.01, *** *p* < 0.001.

To test whether the *osfwl7* mutation affects the accumulation of micronutrient metals in rice, Mn, Cu, Fe, and Zn levels in seedlings with and without Cd treatment were determined. In the absence of Cd, Mn levels in both roots and shoots of *osfwl7* mutants were significantly lower than in those of the WT (Figure 4A,E). Similarly, Cu levels in the roots of *osfwl7* mutants were significantly lower than those of the WT under normal conditions (Figure 4B); however, Cu levels in the shoots of WT and mutants were comparable (Figure 4F). Fe levels in the roots of WT and *osfwl7* mutants grown under normal conditions did not differ (Figure 4C), but its levels in the shoots were significantly lower in the mutants (Figure 4G). Additionally, Zn levels in the roots of *osfwl7a* and in the shoots of both mutants were comparable to those of the WT under normal conditions (Figure 4D,H); however, Zn levels were increased in the roots of *osfwl7b* (Figure 4D).

In the presence of Cd, Cu and Fe levels in the roots of *osfwl7* mutants were lower and higher, respectively, than in those of the WT (Figure 4B,C). Mn levels in the roots were slightly lower in *osfwl7b* but higher in *osfwl7a* under Cd stress (Figure 4A). No significant difference was noted in Zn levels in the roots of the WT and the *osfwl7* mutants under Cd stress (Figure 4D). Additionally, no marked difference was noted in Mn, Cu, Fe, and Zn levels in the shoots under Cd treatment (Figure 4E–H). Collectively, these results suggest that the *osfwl7* mutation affects micronutrient metal accumulation in rice.

### 2.5. osfwl7 Mutation Affects the Expression of Heavy Metal Transporter Genes

**Figure 5:**
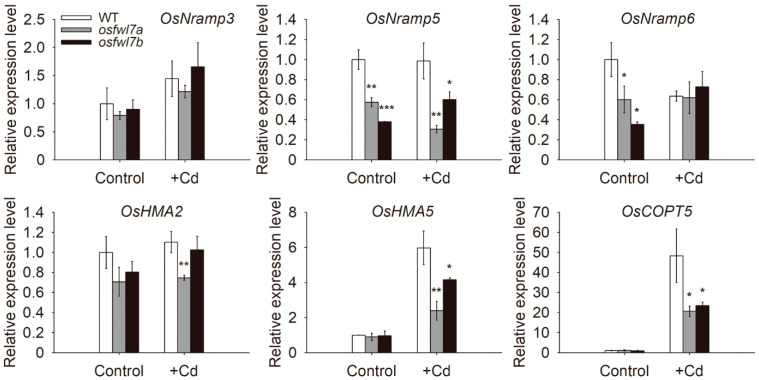
Expression patterns of heavy metal transporter genes in the wildtype (WT) and *osfwl7* mutants determined using RT-qPCR. The genes assayed were as follows: *OsNramp3* (*LOC_Os06g46310*), *OsNramp5* (*LOC_Os07g15370*), *OsNramp6* (*LOC_Os01g31870*), *OsHMA2* (*AB697186*), *OsHMA5* (*LOC_Os04g46940*), and *OsCOPT5* (*LOC_Os05g35050*). The rice *Actin1* gene was used for normalization of gene expression. Error bars indicate the standard deviation of three biological replicates. * *p* < 0.05, ** *p* < 0.01, *** *p* < 0.001.

To elucidate the mechanisms underlying changes in heavy metal concentrations in *osfwl7* mutants, the expression levels of several heavy metal transporter genes in seedling roots of the WT and the mutants were compared. OsNramp5 is the major transporter for Mn and Cd uptake in rice [4,5,6]. The transcript levels of *OsNramp5* were significantly lower in *osfwl7* mutants both under normal and Cd stress conditions (Figure 5). Moreover, OsNramp6 functions as the transporter for Fe and Mn [43]. The transcript levels of *OsNramp6* were lower in the *osfwl7* mutants than in the WT under normal conditions, but comparable under Cd stress (Figure 5). OsCOPT5 is the transporter associated with Cu uptake and redistribution [17], while OsHMA5 is involved in Cu xylem loading [18]. The transcript levels of *OsHMA5* and *OsCOPT5* were significantly lower in *osfwl7* mutants than in the WT under Cd stress (Figure 5). OsHMA2 is involved in the root-to-shoot translocation of Zn and Cd [12,13,14]. The transcript level of *OsHMA2* was significantly lower in the *osfwl7a* mutant than in the WT under Cd stress. Additionally, OsNramp3 functions as a switch for Mn distribution [44]. However, the transcript levels of *OsNramp3* did not significantly differ between the WT and the *osfwl7* mutants both under normal and Cd stress conditions (Figure 5). Together, these results suggest that *osfwl7* mutation affects the expression of several heavy metal transporter genes.

### 2.6. Rice FWL Proteins Interact with Themselves and One Another

**Figure 6:**
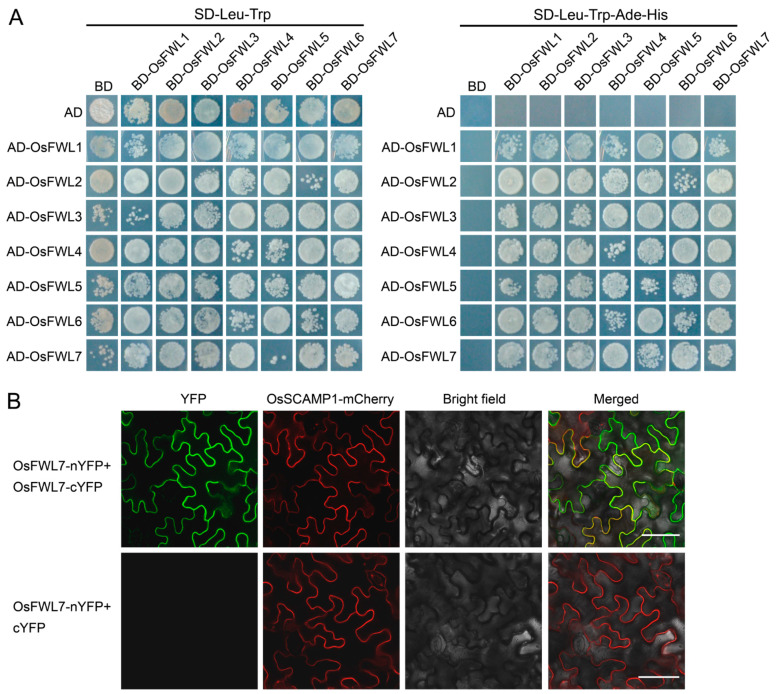
Detection of the interactions of rice FWL proteins. (**A**) Yeast two-hybrid assays showing that all tested rice FWL proteins interact with themselves and one another. Transformed yeast cells were cultured on SD-Leu-Trp control medium and SD-Leu-Trp-Ade-His selective medium. Yeast strains containing the *OsFWL-BD* and empty pGADT7 vectors, the *OsFWL-AD* and empty pGBKT7 vectors, or the empty pGADT7 and pGBKT7 vectors were used as negative controls. AD, activation domain; BD, DNA binding domain. (**B**) Bimolecular fluorescence complementation (BiFC) assays showing self-interaction of OsFWL7 in leaf epidermal cells of *Nicotiana benthamiana*. The OsSCAMP1 protein was fused with mCherry and used as the plasma membrane marker. The YFP fluorescence, mCherry fluorescence, bright field, and merged images are shown. Bar = 50 μm.

In addition to OsFWL7, discussed in this study, six other rice FWL proteins, namely OsFWL1–OsFWL6, have been found to be plasma membrane proteins [33,38]. The plasma membrane-bound plant FWL proteins, such as AtPCR2, OsFWL4, and OsPCR1/FWL5, form homo-oligomers [30,36,38]. To test whether other plasma membrane-bound rice FWL proteins can self-interact, we performed yeast two-hybrid assays and found that all plasma membrane-bound rice FWL proteins interacted with themselves in yeast cells (Figure 6A). Bimolecular fluorescence complementation (BiFC) assays confirmed the self-interaction of OsFWL7 in the cell membrane (Figure 6B). 

We next examined whether the plasma membrane-bound rice FWL proteins can interact with one another using yeast two-hybrid assays and found that all proteins interacted with one another in the yeast cells (Figure 6A). Together, these results indicate that the rice FWL proteins interact with themselves and one another.

### 2.7. Rice FWL Proteins Interact with Membrane Microdomain Marker Proteins

**Figure 7:**
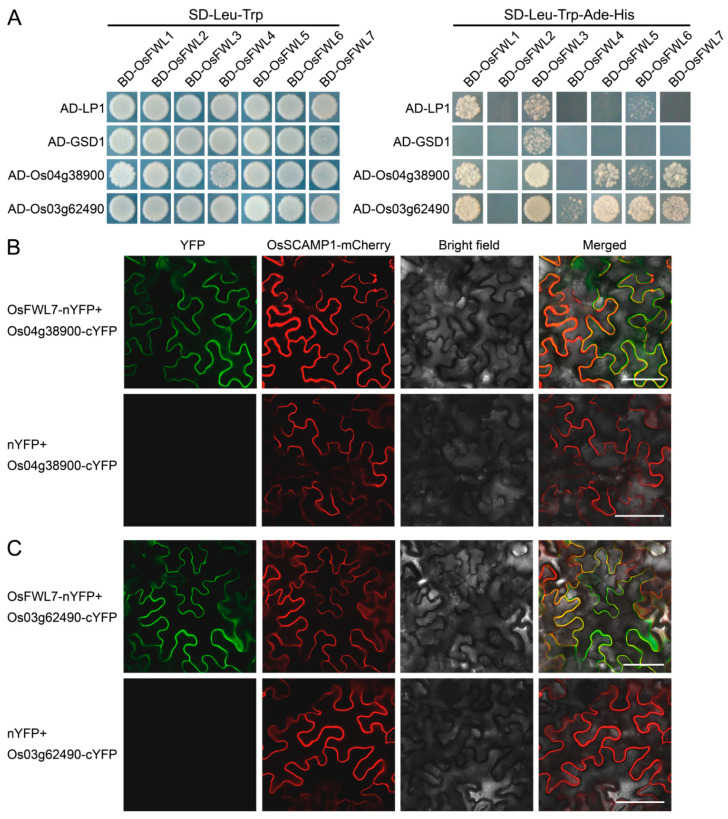
Detection of the interactions of rice FWL proteins with membrane microdomain marker proteins. (**A**) Yeast two-hybrid assays of the interactions between rice FWL proteins and membrane microdomain marker proteins. Transformed yeast cells were cultured on SD-Leu-Trp control medium and SD-Leu-Trp-Ade-His selective medium. AD, activation domain; BD, DNA binding domain. (**B**,**C**) Bimolecular fluorescence complementation (BiFC) assays verify the interaction of OsFWL7 with LOC_Os04g38900 (**B**) and LOC_Os03g62490 (**C**). The OsSCAMP1 protein was fused with mCherry and used as the plasma membrane marker. The YFP fluorescence, mCherry fluorescence, bright field, and merged images are shown. Bar = 50 μm.

The GmFWL1 protein is located in the plasma membrane microdomains [27,28], and remorins and prohibitins are considered the marker proteins of membrane microdomains [45,46]. To test whether the rice FWL proteins are membrane microdomain-associated proteins, the interactions of the seven plasma membrane-bound rice FWL proteins with two remorin family proteins (LP1 and GSD1) [47,48] and two prohibitin family proteins (LOC_Os04g38900 and LOC_Os03g62490) were examined using yeast two-hybrid assays. All tested rice FWL proteins, except OsFWL2, interacted with LOC_Os03g62490 in yeast cells (Figure 7A). Five proteins (OsFWL1, OsFWL3, OsFWL5–OsFWL7) interact with LOC_Os04g38900. However, only OsFWL1, OsFWL3, and OsFWL6 interacted with LP1, and OsFWL3 interacted with GSD1 (Figure 7A). The interactions between OsFWL7 and the two prohibitin family proteins were further verified by BiFC (Figure 7B,C). Collectively, these results indicate that rice FWL proteins interact with membrane microdomain marker proteins.

## 3. Discussion

Cd is a major heavy metal contaminant that is highly toxic to both plants and humans. Previous studies have suggested that plant *FWL* genes play vital roles in the uptake and translocation of Cd [29,31,32,35,36,38]. In the present study, both the uptake and root-to-shoot translocation of Cd were reduced in the *osfwl7* mutants compared with the WT plants under Cd exposure (Figure 3). Similarly, Cd translocation was also decreased in the *OsFWL4*-knockdown plants [36]. When cultured in a liquid medium containing Cd, yeast cells expressing *OsFWL7* accumulated markedly less Cd than the negative controls [36], suggesting that *OsFWL7* inhibits Cd accumulation in cells. Additionally, the expression level of *OsNramp5*, a major transporter involved in Cd uptake, was lower in *osfwl7* mutants than in the WT under Cd treatment (Figure 5). Therefore, *OsFWL7* affects Cd accumulation in rice.

Micronutrient metals, such as Mn, Cu, and Fe, are essential for plant growth and development. Mn levels in both roots and shoots of *osfwl7* mutants were markedly lower than in those of the WT under normal growth conditions (Figure 4A,E). Similarly, the shoot Mn level was significantly reduced in the *OsFWL4*-knockdown plants [36]. The transcript levels of *OsNramp5*, which is also a major transporter for Mn uptake, as well as of another Mn transporter, *OsNramp6*, were lower in the mutants than in the WT under normal conditions (Figure 5). Additionally, Cu levels in the roots and Fe levels in the shoots of *osfwl7* mutants grown under normal conditions were markedly lower than those of the WT (Figure 4B,G). Therefore, *OsFWL7* plays a role in micronutrient metal accumulation in rice.

In this study, the growth of both WT and *osfwl7* mutant plants was severely inhibited following their exposure to 50 μM Cd for 10 days. However, the mutants grew slightly better than the WT (Figure 2), suggesting that the former were less sensitive to Cd stress. This result was consistent with the fact that *osfwl7* mutants accumulated less Cd; thus, suffered from less toxicity of this heavy metal (Figure 3). Interestingly, the root length of *osfwl7* mutants grown under normal conditions was slightly lower than that of the WT, but the shoot dry weight of the mutants was significantly higher (Figure 2). Previous studies suggest that plant FWL proteins can act as the regulators of organ size [19,22,26,34,38]. Hence, *OsFWL7* may also play a role in the regulation of organ development in rice.

Oligomerization of membrane proteins plays important roles in cell processes, such as membrane trafficking and signal transduction [49,50]. Previous studies suggest that the AtPCR2, OsFWL4, and OsPCR1/FWL5 proteins can form homo-oligomers in the plasma membrane to form the pores of the transporter [30,36,38]. In the present study, the seven membrane-bound rice FWL proteins were found to self-interact and interact with one another in the yeast cells (Figure 6A). The self-interaction of OsFWL7 was further confirmed by BiFC assays (Figure 6B). These results indicate that the rice FWL proteins form both homo- and hetero-oligomers in the cell membrane. Such homo- and hetero-oligomerization of proteins has also been observed in plant ammonium transporters [51,52]. 

Numerous studies have suggested that plasma membrane-associated plant FWL proteins perform diverse functions, such as cell division and organ development control, and heavy metal uptake and translocation [24,25]. However, the mechanisms underlying their distinct roles remain elusive. Recent studies have established that the GmFWL1 protein is located in plasma membrane microdomains [27,28], shedding light on the molecular function of plant FWL proteins. Membrane microdomains are sub-compartments of biological membranes and comprise special lipids and proteins [23,45]. They play important roles in diverse biological processes, such as membrane transport and signal transduction [45,53]. Hence, plasma membrane microdomain-localized plant FWL proteins may be involved in transmembrane transportation of metal ions and signaling molecules, thus, affecting metal ion homeostasis and/or organ growth.

Membrane microdomains harbor specific proteins, such as remorins and prohibitins [45,46]. Our yeast two-hybrid assays revealed that six plasma membrane-bound rice FWL proteins interact with the prohibitin family protein LOC_Os03g62490, five interact with the prohibitin family protein LOC_Os04g38900, three interact with the remorin family protein LP1, and one interacts with the remorin family protein GSD1 (Figure 7A). The interactions between OsFWL7 and the two prohibitin family proteins were further verified by BiFC assays (Figure 7B,C). The results obtained indicate that rice FWL proteins interact with membrane microdomain marker proteins and may be located in membrane microdomains, similar to GmFWL1. In fact, OsPCR1/FWL5 was found to be specifically localized to the detergent-resistant membrane [38], which is thought to be similar to a membrane microdomain [53].

## 4. Materials and Methods

### 4.1. Plant Materials and Treatments

The *osfwl7a* and *osfwl7b* mutants were generated previously [37]. The WT rice *O. sativa* L. ssp. *japonica* variety Zhonghua 11 was used as the control. 

Rice seeds were disinfected via treatment with 5% NaClO for 20 min and washed with sterile water. The seeds were then incubated in sterile water for 2 days at 30 °C in the dark. The germinated seeds were grown in distilled water in a growth chamber under a light/dark cycle of 14/10 h and at a day/night temperature of 30 °C/25 °C. On day 3, seedlings with uniform growth were cultured in Kimura B solution for another 3 days before treatment. For the expression analysis of rice *FWL* genes under Cd stress, WT seedlings were grown in Kimura B solution supplemented with different concentrations of Cd for 20 h. Cd was added in the form of CdCl_2_. For the expression analysis of rice heavy metal transporter genes, the WT and mutant seedlings were grown in Kimura B solution with or without 50 μM Cd for 3 days. For tissue-specific expression analysis of *OsFWL7*, different tissues were sampled from the well-grown field plants of Zhonghua 11. All sampled tissues were immediately frozen in liquid nitrogen and stored at −80 °C until assayed.

To analyze Cd tolerance, the WT and mutant seedlings were grown in Kimura B solution containing 50 μM Cd for 10 days. Leaf pigments were measured according to the method described by Arnon [54]. 

### 4.2. Subcellular Localization Analysis

The coding region of *OsFWL7* without the stop codon was amplified, cloned into the pAN580-GFP vector, and transformed into rice protoplasts. The *OsSCAMP1*-mCherry vector was co-transformed and used as the plasma membrane marker [55]. Fluorescence signals were observed using the LSM 700 confocal laser scanning microscope (Carl Zeiss, Jena, Germany). The PCR primers used for the construction of the subcellular localization vector are listed in Table S1.

### 4.3. RNA Isolation and RT-qPCR

Total RNA was isolated using the RNAsimple Total RNA Isolation Kit (Tiangen, Beijing, China). Next, 1 μg of total RNA was reverse-transcribed using the PrimeScript RT reagent Kit with gDNA Eraser (Takara, Dalian, China). RT-qPCR was performed using the CFX Connect Real-Time PCR system (Bio-Rad, Hercules, CA, USA). Each reaction mixture had a final volume of 25 μL, containing 2 μL of cDNA template, 12.5 μL of TB Green Premix Ex Taq II (Takara), and 0.4 μM gene-specific primers. The PCR cycle was as follows: initial incubation at 95 °C for 30 s, followed by 40 cycles at 95 °C for 5 s and at 60 °C for 34 s. We then performed melting curve analysis of amplicons to determine the specificity of PCR. Rice *Actin1* or ubiquitin genes were used for data normalization. We used the 2^−ΔΔCT^ method to calculate relative expression levels of target genes. Primers used for RT-qPCR are listed in Table S1.

### 4.4. Yeast Two-Hybrid Assay

The coding regions of the rice *FWL* genes were cloned into the pGBKT7 bait vector and pGADT7 prey vector. The two remorin and two prohibitin genes were cloned into the pGADT7 vector. The bait and prey vectors were co-transformed into yeast strain AH109 using the Yeastmaker Yeast Transformation System 2 kit (Clontech, Dalian, China). After culturing on SD-Leu-Trp medium for 2 days, the interactions between the bait and prey were detected on selective SD-Leu-Trp-Ade-His medium. Yeast strains harboring the *OsFWL-BD* and empty pGADT7 vectors, the *OsFWL-AD* and empty pGBKT7 vectors, or the empty pGADT7 and pGBKT7 vectors were used as negative controls. All assays were repeated at least twice. The PCR primers used for the construction of yeast hybridization vectors are listed in Table S1.

### 4.5. BiFC Assay

The coding sequence of *OsFWL7* was cloned into the p2YN and p2YC vectors to generate *OsFWL7-YN* and *OsFWL7-YC* constructs, respectively. The coding sequences of the two prohibitin genes were cloned into the p2YC vector. The constructs were transformed into *Agrobacterium tumefaciens* strain EHA105 and transfected *Nicotiana benthamiana* leaves. The fluorescence was monitored using the LSM 700 confocal laser scanning microscope (Carl Zeiss). The PCR primers used for the construction of BiFC vectors are listed in Table S1.

### 4.6. Measurement of Heavy Metal Levels

Heavy metal levels in different tissues were determined according to the methods described by Zhang et al. [56]. In brief, the roots of hydroponically cultured seedlings were soaked in 20 mM EDTA for 15 min and washed with deionized water. The root and shoot samples were dried at 80 °C for 3 days and ground to a fine powder using an analytical mill (Cole-Parmer, Vernon Hills, IL, USA). Then, the samples (0.5 g) were digested with HNO_3_ and H_2_O_2_ in a microwave digestion instrument (MARS 5; CEM, Matthews, NC, USA). Cd and micronutrient metal levels were measured using inductively coupled plasma optical emission spectrometry (ICP-OES, Thermo Scientific iCAP 6300; Thermo Fisher Scientific, Grand Island, NY, USA).

### 4.7. Statistical Analysis

All data are presented as mean and standard deviation of replicates. The statistical significance of differences in means was evaluated using Student’s *t*-test. Column charts were produced using SigmaPlot 10.0.

## 5. Conclusions

In summary, *OsFWL7* encodes a plasma membrane protein that regulates Cd and micronutrient metal accumulation in rice. The rice FWL proteins interact with themselves and one another and may be located in membrane microdomains.

## Data Availability

All data are presented as figures and Supplementary Materials, which are included in this paper.

## Supplementary Materials

The following are available online at https://www.mdpi.com/article/10.3390/ijms222212583/s1.
