# Supplementary material for: Mutation in OsFWL7 Affects Cadmium and Micronutrient Metal Accumulation in Rice
Source: Int J Mol Sci. 2021 Nov 22;22(22):12583. doi: 10.3390/ijms222212583 (PMC8624461; doi:10.3390/ijms222212583)
Supplement: Supplementary file 1 [file ijms-22-12583-s001.zip › Supplementary materials-revised.pdf]

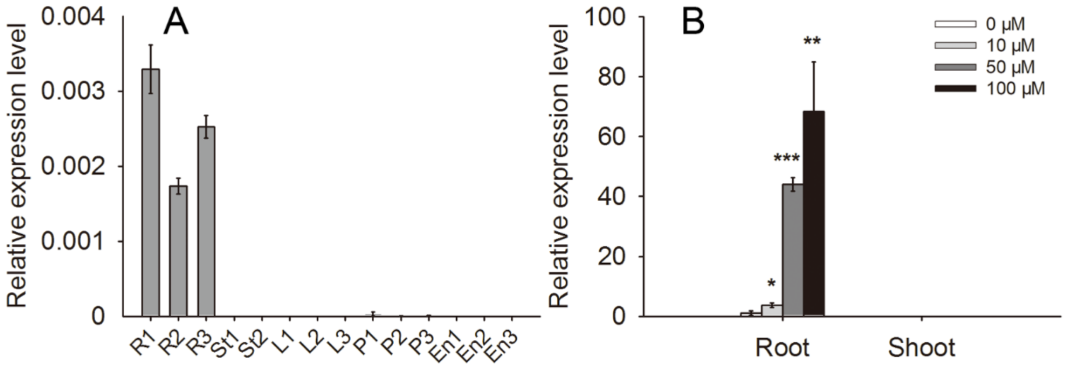

Figure S1. RT-qPCR results of *OsFWL7* gene in different tissues and under Cd treatments using the rice ubiquitin gene as a reference. (**A**) RT-qPCR results of *OsFWL7* in 14 tissue samples of *Oryza sativa* L. ssp. *japonica* variety Zhonghua 11. The tissues used were as follows: seedling, tillering, and heading stage roots (R1–R3); jointing and heading stage stems (St1 and St2); seedling, tillering, and heading stage leaves (L1–L3); 5-, 15-, and 20-cm panicles (P1–P3); and endosperms 5, 14, and 21 days after pollination (En1–En3). Error bars indicate the standard deviation of three technical replicates. (**B**) RT-qPCR results of *OsFWL7* under treatment with Cd of different concentrations. Error bars indicate the standard deviation of three biological replicates. *$p < 0.05$, **$p < 0.01$, ***$p < 0.001$.

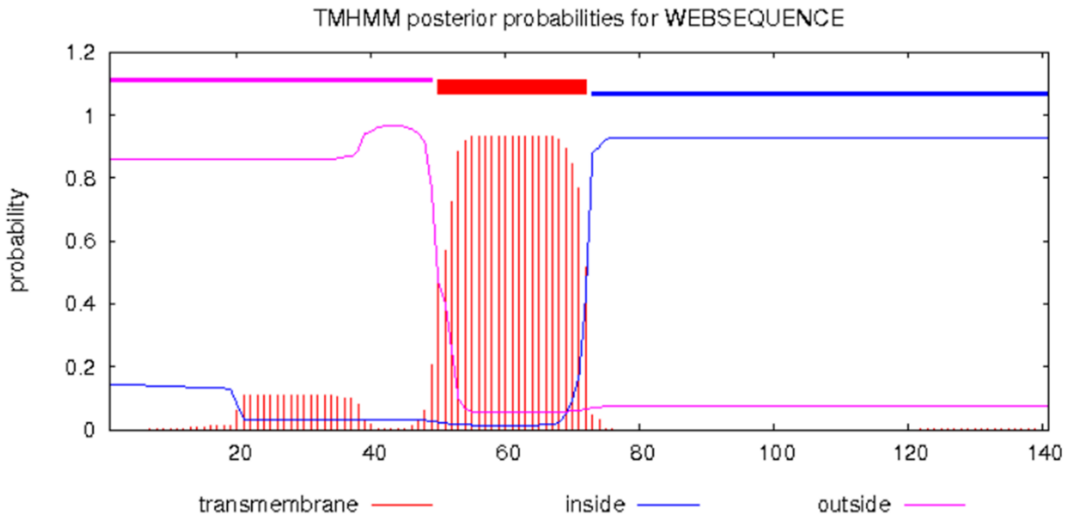

Figure S2. Prediction of transmembrane helices in OsFWL7 using TMHMM Server v. 2.0.

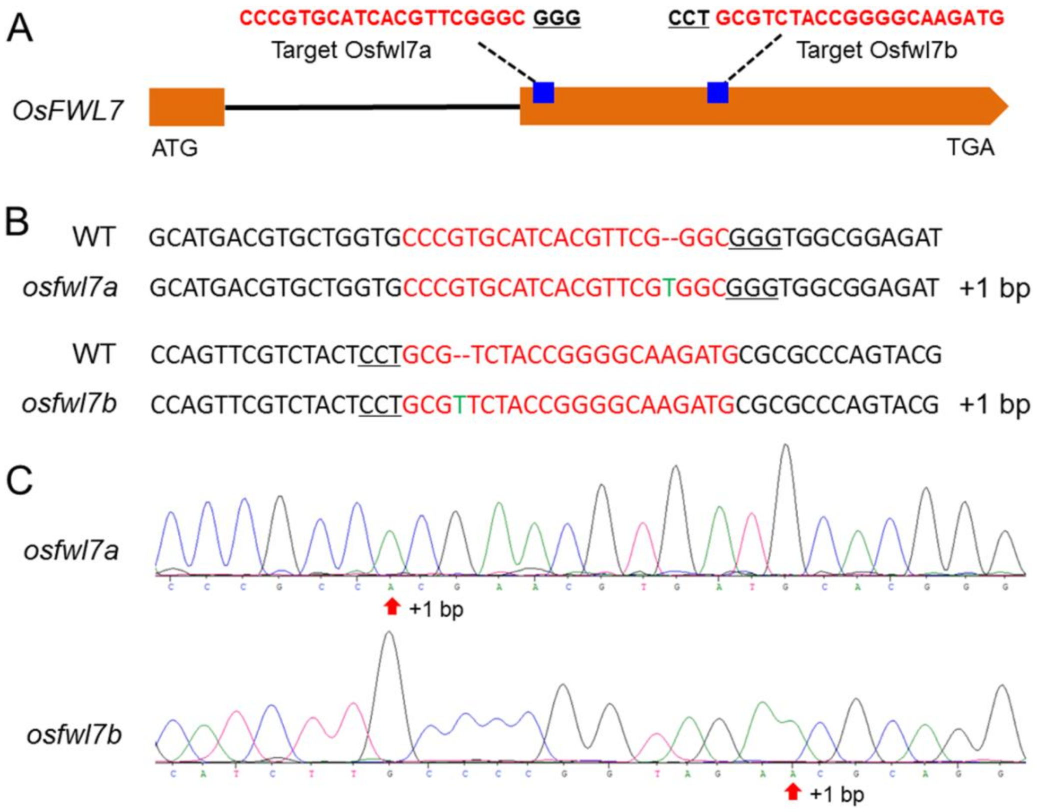

Figure S3. Molecular identification of *OsFWL7* mutants generated through CRISPR/Cas9 genome editing. (**A**) Schematic diagram of the two target sites in *OsFWL7*. (**B**) Genotypes of *osfwl7a* and *osfwl7b* mutants. (**C**) Sequencing chromatograms of *osfwl7a* and *osfwl7b* mutants.

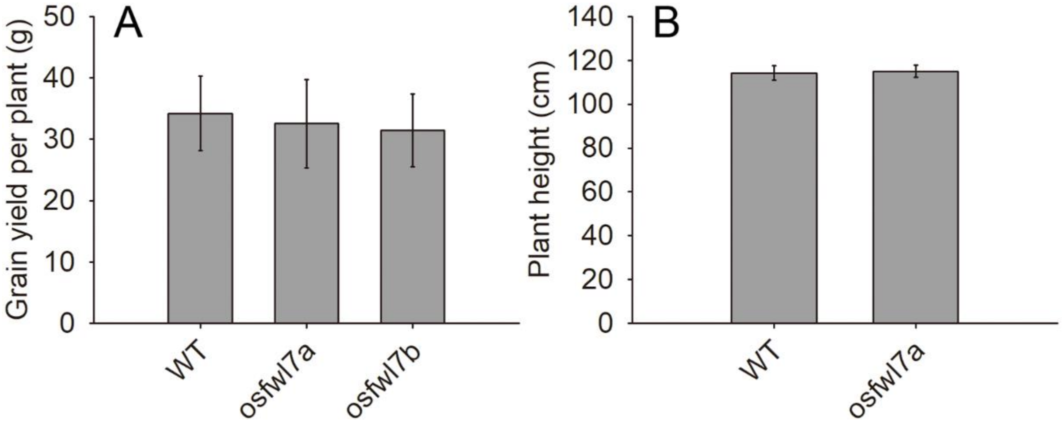

Figure S4. The grain yield per plant (**A**) and plant height (**B**) of the wildtype (WT) and *osfwl7* mutants. The values in (**A**) and (**B**) are means ± standard deviation of 10 and 15 plants, respectively.

Table S1. Primers used in this study.

Experiment	Primer name	Sequence (5′ to 3′)
Subcellular localization	OsFWL7SL-F	CGGAGCTAGCTCTAGAATGGCCAAGCCAAGCGCCGC
	OsFWL7SL-R	TGCTCACCATGGATCCACGGCCCATGTGCTGCACGG
Yeast two-hybrid assay	OsFWL1AD-F	GGAGGCCAGTGAATTCATGTATCCCTCCGCCCCTCC
	OsFWL1AD-R	CGAGCTCGATGGATCCCTACCTCATCATGCCGACCG
	OsFWL2AD-F	GGAGGCCAGTGAATTCATGTACTCGAAACCGGAGGA
	OsFWL2AD-R	CGAGCTCGATGGATCCTCAGCGTGTCATCCCGGGGA
	OsFWL3AD-F	GGAGGCCAGTGAATTCATGCAGGACCAGGCCGCCCC
	OsFWL3AD-R	CGAGCTCGATGGATCCTCAGCGTGTCATCCCAGGGT
	OsFWL4AD-F	GGAGGCCAGTGAATTCATGGCGAGGCCGCAACACAA
	OsFWL4AD-R	CGAGCTCGATGGATCCTTAGCGTCGCATCGGCGGGT
	OsFWL5AD-F	GGAGGCCAGTGAATTCATGTATCCCCCTGATCCGTC
	OsFWL5AD-R	CGAGCTCGATGGATCCTTATGAGGGAGTTGTCATTC
	OsFWL6AD-F	GGAGGCCAGTGAATTCATGGCCAAGCCAAGCGCCGC
	OsFWL6AD-R	CGAGCTCGATGGATCCTTAGCGGCCCATGTACTGCA
	OsFWL7AD-F	GGAGGCCAGTGAATTCATGGCCAAGCCAAGCGCCGC
	OsFWL7AD-R	CGAGCTCGATGGATCCTTAACGGCCCATGTGCTGCA
	LP1AD-F	GGAGGCCAGTGAATTCATGGAGAGAATGCGGTGTGT
	LP1AD-R	CGAGCTCGATGGATCCCTAGAAGGCATGGCAAGTGA
	GSD1AD-F	GGAGGCCAGTGAATTCATGGAGTATGAAAGGATTCA
	GSD1AD-R	CGAGCTCGATGGATCCCTAGTGTTGACAGAAGCAAC
	Os04g38900AD-F	GGAGGCCAGTGAATTCATGGCCGGCGGTCCCGCGGC
	Os04g38900AD-R	CGAGCTCGATGGATCCTCACCGGCCGAACCCGGCAG
	Os03g62490AD-F	GGAGGCCAGTGAATTCATGAACTTCAAGGGCGCGAA
	Os03g62490AD-R	CGAGCTCGATGGATCCTCACTTCTTGTTTTTGTTGT
	OsFWL1BD-F	CATGGAGGCCGAATTCATGTATCCCTCCGCCCCTCC
	OsFWL1BD-R	GCAGGTCGACGGATCCCTACCTCATCATGCCGACCG
	OsFWL2BD-F	CATGGAGGCCGAATTCATGTACTCGAAACCGGAGGA
	OsFWL2BD-R	GCAGGTCGACGGATCCTCAGCGTGTCATCCCGGGGA
	OsFWL3BD-F	CATGGAGGCCGAATTCATGCAGGACCAGGCCGCCCC
	OsFWL3BD-R	GCAGGTCGACGGATCCTCAGCGTGTCATCCCAGGGT
	OsFWL4BD-F	CATGGAGGCCGAATTCATGGCGAGGCCGCAACACAA
	OsFWL4BD-R	GCAGGTCGACGGATCCTTAGCGTCGCATCGGCGGGT
	OsFWL5BD-F	CATGGAGGCCGAATTCATGTATCCCCCTGATCCGTC
	OsFWL5BD-R	GCAGGTCGACGGATCCTTATGAGGGAGTTGTCATTC
	OsFWL6BD-F	CATGGAGGCCGAATTCATGGCCAAGCCAAGCGCCGC
	OsFWL6BD-R	GCAGGTCGACGGATCCTTAGCGGCCCATGTACTGCA
	OsFWL7BD-F	CATGGAGGCCGAATTCATGGCCAAGCCAAGCGCCGC
	OsFWL7BD-R	GCAGGTCGACGGATCCTTAACGGCCCATGTGCTGCA
BiFC assay	FWL7YN-F	CATTTACGAACGATAGTTAATTAAATGGCCAAGCCAAGCGCCGC
	FWL7YN-R	CACTGCCACCTCCTCCACTAGTACGGCCCATGTGCTGCACGG
	FWL7YC-F	CATTTACGAACGATAGTTAATTAAATGGCCAAGCCAAGCGCCGC
	FWL7YC-R	CACTGCCACCTCCTCCACTAGTACGGCCCATGTGCTGCACGG

	Os04g38900YC-F	CATTTACGAACGATAGTTAATTAAATGGCCGGCGGTCCCGCGGC
	Os04g38900YC-R	CACTGCCACCTCCTCCACTAGTCCGGCCGAACCCGGCAGCGT
	Os03g62490YC-F	CATTTACGAACGATAGTTAATTAAATGAACTTCAAGGGCGCGAA
	Os03g62490YC-R	CACTGCCACCTCCTCCACTAGTCTTCTTGTTTTTGTTGTCCA
RT-qPCR analysis	OsFWL1F	GATTTCCTCGTCCATTTCTGC
	OsFWL1R	GTTCGCCATCTTCGTCTACCT
	OsFWL2F	AGCCAGATTTTTCTTGCGG
	OsFWL2R	GGAACCGAGGGAAACCAA
	OsFWL3F	GGCACGCAAACATGGAGAA
	OsFWL3R	AGAGAAGACAACGACCGAGCA
	OsFWL4F	ATGACTGCGAAGTTTGTTGCT
	OsFWL4R	CCCCTCGACACGATCTCC
	OsFWL6F	GGGGAAGAAAAGTAGGAGGAGA
	OsFWL6R	CAGCACGTCAAGCAGCATAG
	OsFWL7F	GGCGAAGGAAATTAAAGGAAAG
	OsFWL7R	GGTAGACGCAGGAGTAGACGAA
	OsNramp3F	CACACAAATTCAAAAGCCATTTCTG
	OsNramp3R	CGATAGACCGTTGTGGAGAAGC
	OsNramp5F	CAGCAGCAGTAAGAGCAAGATG
	OsNramp5R	GTGCTCAGGAAGTACATGTTGAT
	OsNramp6F	CATAGCTGCGATTGTACTATCATTTG
	OsNramp6R	CGCGATTCGGATAATAGATGTTG
	OsHMA2F	CATAGTGAAGCTGCCTGAGATC
	OsHMA2R	GATCAAACGCATAGCAGCATCG
	OsHMA5F	AAGGTGGAGAGTATAATGGTGAC
	OsHMA5R	CCTTCCGGCCGACTGAAGTTC
	OsCOPT5F	GCTGTCTCGCTCGTCATGGT
	OsCOPT5R	CGCACACACAAAACATCAACAA
	ActinRT-F	CTTGGCATCTCTCAGCACATT
	ActinRT-R	TTGGCTTAGCATTCTTGGGT
	Ubiquitin-F	AACCAGCTGAGGCCCAAGA
	Ubiquitin-R	ACGATTGATTTAACCAGTCCATGA
